# Supplementary material for: Analysis of relative abundances with zeros on environmental gradients: a multinomial regression model
Source: PeerJ. 2018 Sep 27;6:e5643. doi: 10.7717/peerj.5643 (PMC6164550; doi:10.7717/peerj.5643)
Supplement: Supplemental Information 2 [file peerj-06-5643-s002.pdf]

Analysis of relative abundances with zeros on environmental gradients: a multinomial regression model. Supplemental information

Fiona Chong and Matthew Spencer

August 17, 2018

S1 Compositional algebra

Ecologists make frequent use of some aspects of vector algebra in $\mathbb{R}^n$, a mathematical system which emerged gradually in the late 19th century, primarily driven by the need to solve three-dimensional physical problems in fields such as electricity (Crowe, 1994). In community ecology, the main application of vectors is the representation and manipulation of the abundances of more than one species simultaneously. For such vectors, the operations of addition and scalar multiplication have obvious biological meanings. However, ecologists make little explicit use of the more abstract concept of a real vector space, defined only by the axioms it satisfies, rather than the types of objects involved. This concept, now important in many areas of mathematics, emerged around the same time as vector algebra (Dorier, 1995). A real vector space is a set of objects (vectors) with a binary operation ('addition'), and a scalar operation ('scalar multiplication') by which real numbers act on the objects (Fraleigh and Beauregard,

1995, section 3.1). The addition operation satisfies the familiar algebraic axioms of closure, associativity, commutativity, and the existence of an identity element and of inverse elements. The scalar multiplication operation satisfies the familiar algebraic axioms of closure, distributivity, associativity, and has 1 as the multiplicative identity. This more general concept might be useful in ecology because the ordinary definitions of addition and scalar multiplication for Euclidean vectors do not satisfy the vector space axioms when applied to relative abundances. For example, let $\mathbf{a} = (1/3, 1/3, 1/3)^T$ be a relative abundance vector (throughout, we work with column vectors, so T denotes transpose). Then neither $\mathbf{a} + \mathbf{a}$ nor $2\mathbf{a}$ is a relative abundance vector, so the axiom of closure is not satisfied.

There are in fact operations corresponding to addition and scalar multiplication that make sense for compositions. For a vector of s positive numbers $\mathbf{x}$, let the closure $C(\mathbf{x})$ of $\mathbf{x}$ be defined by

$$C(\mathbf{x}) = \frac{1}{\sum_{i=1}^{s} x_i}\mathbf{x}$$

(Aitchison, 1986, p. 31). Now if $\mathbf{a}, \mathbf{b}$ are s-part compositions, then let the perturbation $\oplus$ of $\mathbf{b}$ by $\mathbf{a}$ be defined by

$$\mathbf{a} \oplus \mathbf{b} = C(a_1 b_1, a_2 b_2, \ldots, a_s b_s)$$

(Aitchison, 1986, p. 42). Also, if $a \in \mathbb{R}$, then the powering $\odot$ of $\mathbf{b}$ by a is defined by

$$a \odot \mathbf{b} = C(b_1^a, b_2^a, \ldots, b_s^a)$$

(Aitchison, 1986, p. 120). The set of s-part compositions with the binary operation of perturbation (corresponding to 'addition') and the scalar operation of powering (corresponding to 'scalar multiplication') satisfies the vector space axioms (Billheimer et al., 2001). Now for any

two compositions **a** and **b**, we can transform **a** into **b** by the closure of the unequal scaling

$$\begin{aligned}\mathbf{b} &= C\left(\frac{b_1}{a_1}a_1, \frac{b_2}{a_2}a_2, \ldots, \frac{b_s}{a_s}a_s\right) \\ &= \mathbf{b} \oplus ((-1) \odot \mathbf{a}) \oplus \mathbf{a}.\end{aligned}$$

We can thus define the compositional difference $\mathbf{b} \ominus \mathbf{a}$ as

$$\mathbf{b} \ominus \mathbf{a} = \mathbf{b} \oplus ((-1) \odot \mathbf{a}) = C\left(\frac{b_1}{a_1}, \frac{b_1}{a_1}, \ldots, \frac{b_s}{a_s}\right). \quad \text{(S1)}$$

This is the only way to define the difference between two compositions, under either one of two additional conditions (Aitchison, 1992): that the difference must not depend on changes of units for individual components, or that the ith component of the transformation from one composition to another must depend only on the ith component of the compositions.

S2 The measurement of dissimilarity between relative abundance vectors

Analyses of community dissimilarity matrices can be misleading if the measure of dissimilarity is not perturbation invariant. Consider a series of J communities along an environmental gradient, with compositions $\boldsymbol{\rho}_1, \boldsymbol{\rho}_2, \ldots, \boldsymbol{\rho}_J$. Suppose that the communities are spaced so that the ratios of relative abundances for each species in successive communities are constant, in other words $\rho_{i,j}/\rho_{i,j+1} = a_i$, where a_i is a constant, for each species $i \in \{1, 2, \ldots, s\}$ and for each community $j \in \{1, 2, \ldots, J-1\}$. Since relative abundances, by definition, are meaningful only in relative terms, there has been the same amount of change in the relative abundance of each species between each pair of communities $j, j+1$. This implies that a meaningful measure of dissimilarity

between adjacent pairs of communities must be constant. From the definition of compositional difference (Equation S1), $\boldsymbol{\rho}_{j+1} \ominus \boldsymbol{\rho}_j = \mathbf{a}$, where $\mathbf{a} = (a_1, a_2, \ldots, a_s)$ is a constant perturbation. Then we can write $\boldsymbol{\rho}_{j+1} = \mathbf{a} \oplus \boldsymbol{\rho}_j$, and $\boldsymbol{\rho}_{j+2} = \mathbf{a} \oplus \boldsymbol{\rho}_{j+1}$, and we require that $d(\boldsymbol{\rho}_j, \boldsymbol{\rho}_{j+1}) = d(\mathbf{a} \oplus \boldsymbol{\rho}_j, \mathbf{a} \oplus \boldsymbol{\rho}_{j+1})$. In general, any meaningful dissimilarity measure d for compositions must satisfy the perturbation invariance property $d(\boldsymbol{\rho}_1, \boldsymbol{\rho}_2) = d(\mathbf{a} \oplus \boldsymbol{\rho}_1, \mathbf{a} \oplus \boldsymbol{\rho}_2)$ for all compositions $\boldsymbol{\rho}_1, \boldsymbol{\rho}_2, \mathbf{a}$. Most of the popular measures of community dissimilarity are not perturbation invariant, and are therefore misleading. For example, let $\boldsymbol{\rho}_1 = (\frac{1}{6}, \frac{1}{3}, \frac{1}{2})^T$, $\boldsymbol{\rho}_2 = (\frac{1}{2}, \frac{1}{3}, \frac{1}{6})^T$, $\mathbf{a} = (\frac{1}{3}, \frac{1}{6}, \frac{1}{2})^T$. Then using `vegdist(method = ‘‘bray’’)` in the R package `vegan 2.4-3` (Oksanen et al., 2017), the Bray-Curtis dissimilarity between $\boldsymbol{\rho}_1$ and $\boldsymbol{\rho}_2$ is 0.333 to three decimal places, but the Bray-Curtis dissimilarity between $\mathbf{a} \oplus \boldsymbol{\rho}_1$ and $\mathbf{a} \oplus \boldsymbol{\rho}_2$ is 0.420 to three decimal places. Other popular measures of community dissimilarity are shown not to be perturbation invariant (in the context of temporal change) in Spencer (2015, Appendix B). In contrast, the Aitchison distance (Aitchison, 1992) is a well-established perturbation-invariant measure of dissimilarity between composition. Thus, analyses of dissimilarity between relative abundances should be based on Aitchison distance, rather than the currently-popular measures of community dissimilarity.

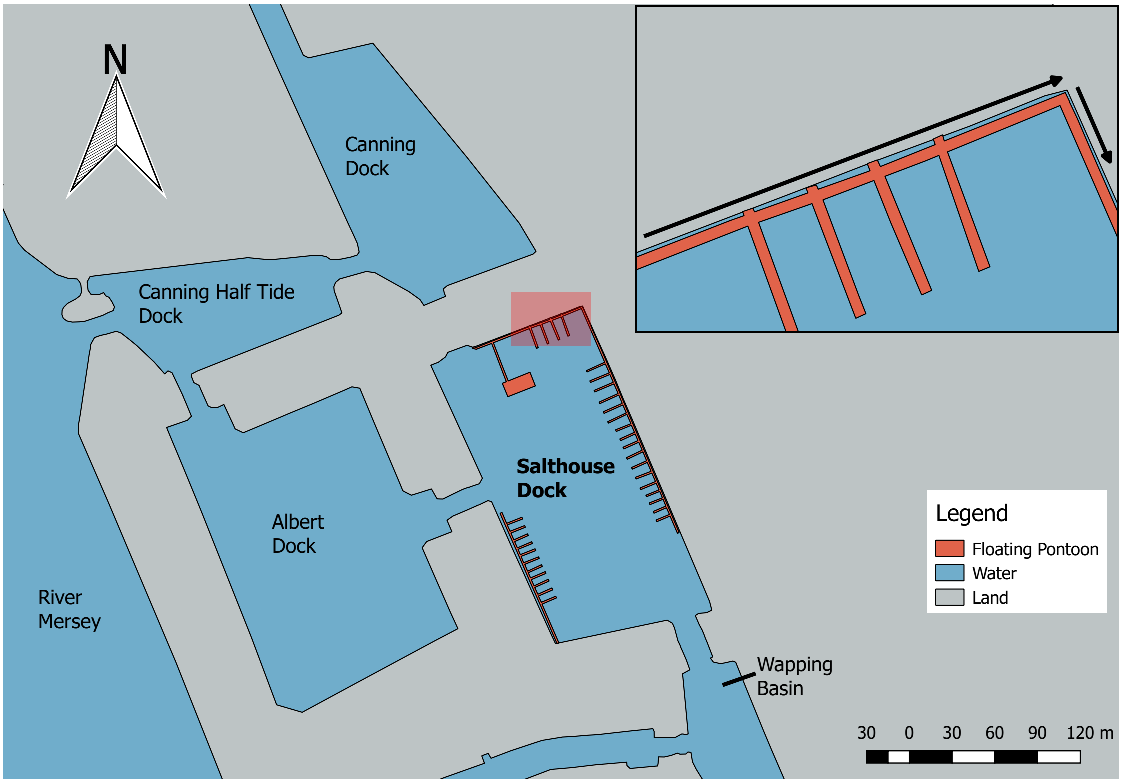

Figure S1: Map of the study site, Salthouse Dock, and its connections to the River Mersey. The floating pontoons allow access to the section of dock wall covered by the transects, which is indicated by the arrows on the inset. Figure drawn based on data from OpenStreetMap (OpenStreetMap contributors, 2018).

S3 Estimation methods

We fitted the model described in the main text using Bayesian estimation, via the NUTS algorithm (Hoffman and Gelman, 2014) in Stan 2.16.0, with the `rstan` package (version 2.15.1) and R version 3.4.1 (R Core Team, 2017). In order to speed up convergence by reducing posterior correlations, we actually used the orthogonal polynomial of order 2 for depth as the explanatory variable, and then obtained the coefficients for depth and squared depth by transformation, as described in Kennedy and Gentle (1980, pp. 342-345). We specified weak independent Cauchy(0, 2.5) priors on β_0 and the coefficients of the orthogonal polynomial of depth, and weak independent half-Cauchy(0, 2.5) priors on the standard deviations of ε. We chose an LKJ prior with scale parameter $\eta = 2$ on the correlation matrix $\mathbf{\Omega}$ of ε (Lewandowski et al., 2009), for which the prior density is proportional to $\det \mathbf{\Omega}$. This means that in the prior, the modal correlation matrix is the identity matrix, but the density is not very concentrated around this mode (Stan Development Team, 2017, section 60.1). We parameterized the correlation matrix using its Cholesky factor. We ran four independent chains, with 5000 warmup and 5000 sampling iterations. We checked that the chains had approximately converged by inspection of trace plots and potential scale reduction statistics (which were less than 1.0015 for all parameters). Effective sample size was at least 1950 for all parameters.

S4 Simulation study

We checked the performance of the estimation method on 100 simulated data sets, generated under the model using the posterior mean estimates from the real data (Tables S1 and S2). We fitted the model to each data set as described above. For almost all parameters (Tables S3 and S4), nominal 95% credible intervals contained the true parameter values 90 to 100 times out of 100. The exceptions were element 3 of the coefficient for squared depth effect, and elements

(6, 6), (7, 7), (8, 1), (8, 6) and (8, 8) of the Cholesky factor of the covariance matrix, where the nominal 95% credible intervals contained the true parameters less often than required.

Table S1: Coefficients β_0 (intercept), β_1 (depth), β_2 (squared depth) from the regression model described in the main text. Values are posterior means and 95% highest posterior density credible intervals in the 8 isometric logratio coordinates corresponding to 9 taxa, with the default basis from the R package compositions. Note that these coordinates have no simple interpretation in terms of the 9 taxa.

β_0	β_1	β_2
-1.11 (-1.30, -0.94)	-0.01 (-0.14, 0.11)	-0.62 (-0.79, -0.45)
0.50 (0.35, 0.65)	-0.10 (-0.20, 0.01)	-0.39 (-0.52, -0.24)
-3.00 (-3.45, -2.56)	-2.69 (-3.17, -2.24)	0.72 (0.29, 1.11)
1.04 (0.86, 1.23)	0.15 (-0.00, 0.31)	-0.24 (-0.40, -0.08)
-3.06 (-3.52, -2.64)	0.59 (0.30, 0.89)	0.39 (0.06, 0.71)
-2.14 (-2.64, -1.66)	1.11 (0.71, 1.50)	-0.17 (-0.56, 0.19)
-7.91 (-11.56, -5.17)	2.46 (0.05, 4.94)	0.56 (-0.56, 2.14)
-3.16 (-4.54, -1.90)	0.07 (-0.38, 0.58)	-0.04 (-0.60, 0.49)

Table S2: Cholesky factor of covariance matrix $\mathbf{\Sigma}$ from the regression model described in the main text. Values are posterior means and 95% highest posterior density credible intervals in the 8 isometric logratio coordinates corresponding to 9 taxa, with the default basis from the R package compositions. Note that these coordinates have no simple interpretation in terms of the 9 taxa.

0.57 (0.46, 0.69)							
-0.15 (-0.26, -0.04)	0.40 (0.32, 0.49)						
-0.22 (-0.53, 0.08)	-0.43 (-0.73, -0.12)	1.04 (0.80, 1.32)					
-0.02 (-0.16, 0.13)	-0.00 (-0.15, 0.14)	0.07 (-0.09, 0.23)	0.55 (0.43, 0.67)				
-0.01 (-0.23, 0.18)	0.01 (-0.19, 0.23)	-0.08 (-0.36, 0.11)	-0.01 (-0.21, 0.19)	0.30 (0.00, 0.69)			
-0.34 (-0.67, -0.01)	0.25 (-0.09, 0.57)	-0.06 (-0.45, 0.31)	-0.05 (-0.38, 0.29)	0.14 (-0.50, 0.72)	0.84 (0.47, 1.21)		
-0.46 (-1.44, 0.50)	-0.43 (-1.46, 0.57)	-0.09 (-1.37, 1.10)	0.60 (-0.43, 1.77)	-0.08 (-1.44, 1.32)	-0.23 (-1.42, 1.01)	1.51 (0.47, 2.87)	
0.49 (-0.11, 1.17)	0.11 (-0.51, 0.75)	-0.22 (-0.90, 0.44)	-0.08 (-0.73, 0.57)	0.06 (-0.98, 1.07)	0.45 (-0.36, 1.32)	0.24 (-0.77, 1.19)	1.01 (0.31, 1.79)

Table S3: Percentage of simulated data sets for which nominal 95% credible intervals contained true parameter values for coefficients β_0 (intercept), β'_1 (linear term in orthogonal polynomial for depth) and β'_2 (quadratic term in orthogonal polynomial for depth). Rows are the 8 ilr components corresponding to 9 taxa, with the default basis from the R package compositions. Note that these coordinates have no simple interpretation in terms of the 9 taxa.

β_0	β'_1	β'_2
98	96	94
94	99	94
98	94	83
97	98	94
94	93	93
95	97	97
98	90	100
96	100	100

Table S4: Percentage of simulated data sets for which nominal 95% credible intervals contained true parameter values for Cholesky factor of error covariance matrix $\mathbf{\Sigma}$. Rows and columns are the 8 ilr components corresponding to 9 taxa, with the default basis from the R package compositions. Note that these coordinates have no simple interpretation in terms of the 9 taxa.

	1	2	3	4	5	6	7	8
1	98	0	0	0	0	0	0	0
2	95	94	0	0	0	0	0	0
3	96	93	99	0	0	0	0	0
4	97	96	99	93	0	0	0	0
5	100	100	100	100	99	0	0	0
6	97	91	99	99	100	86	0	0
7	92	100	100	92	100	100	84	0
8	80	100	99	100	100	89	100	78

Posterior distributions of parameters from simulated data were generally centred not too far from the true values, although there was more variation in location for intercept and depth coefficients (Figure S2) than for elements of the Cholesky factor of the error covariance matrix (Figure S3). However, the posterior distributions of parameters for ilr components 7 and 8 had very long tails (Figures S2 and S3, bottom two rows).

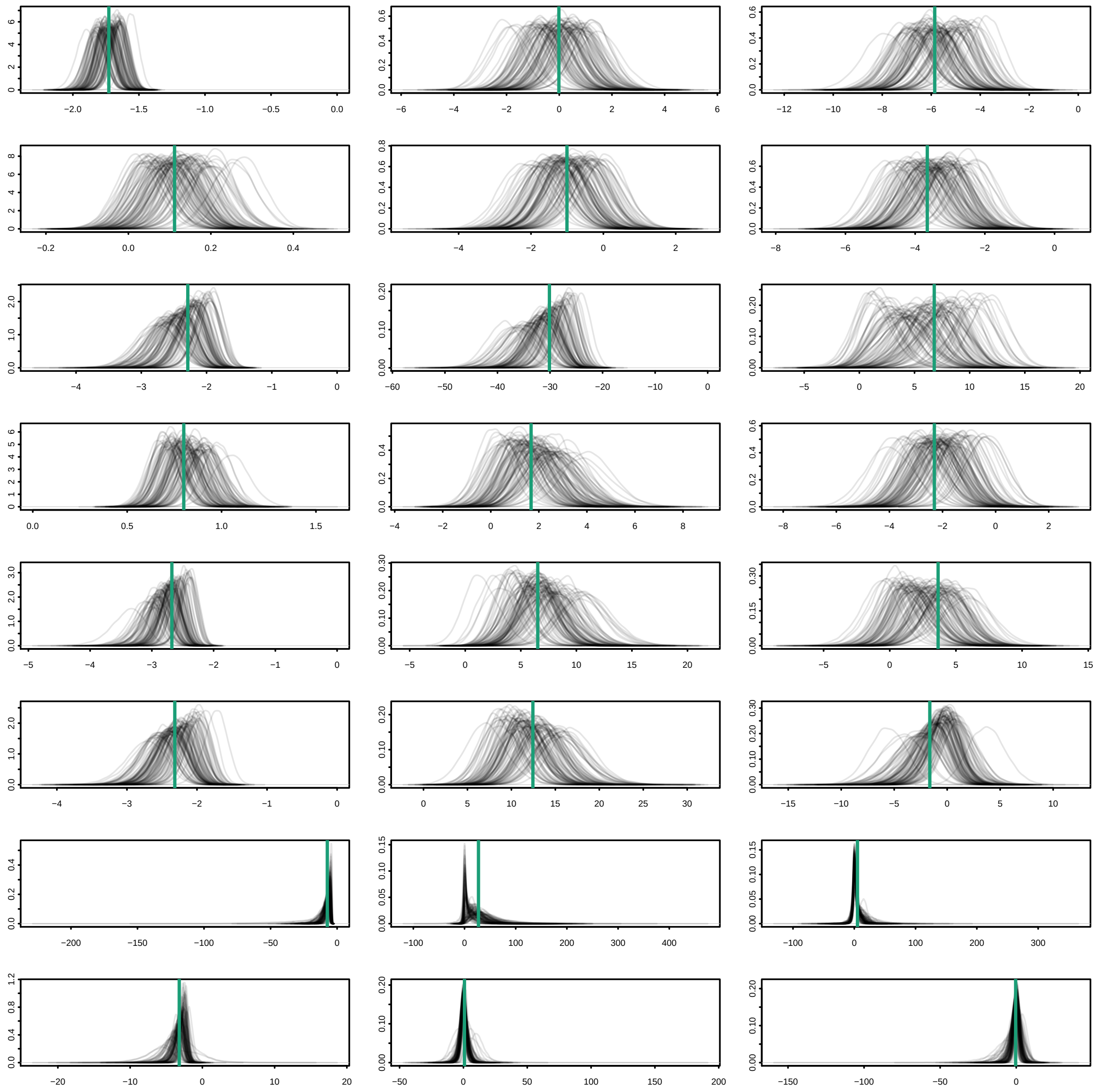

Figure S2: Posterior distributions (black lines) from 100 simulated data sets of estimated coefficients β_0 (intercept, first column), β_1' (linear term in orthogonal polynomial for depth, second column) and β_2' (quadratic term in orthogonal polynomial for depth, third column). Vertical green lines: true parameter values. Rows are the 8 ilr components corresponding to 9 taxa, with the default basis from the R package compositions. Note that these coordinates have no simple interpretation in terms of the 9 taxa.

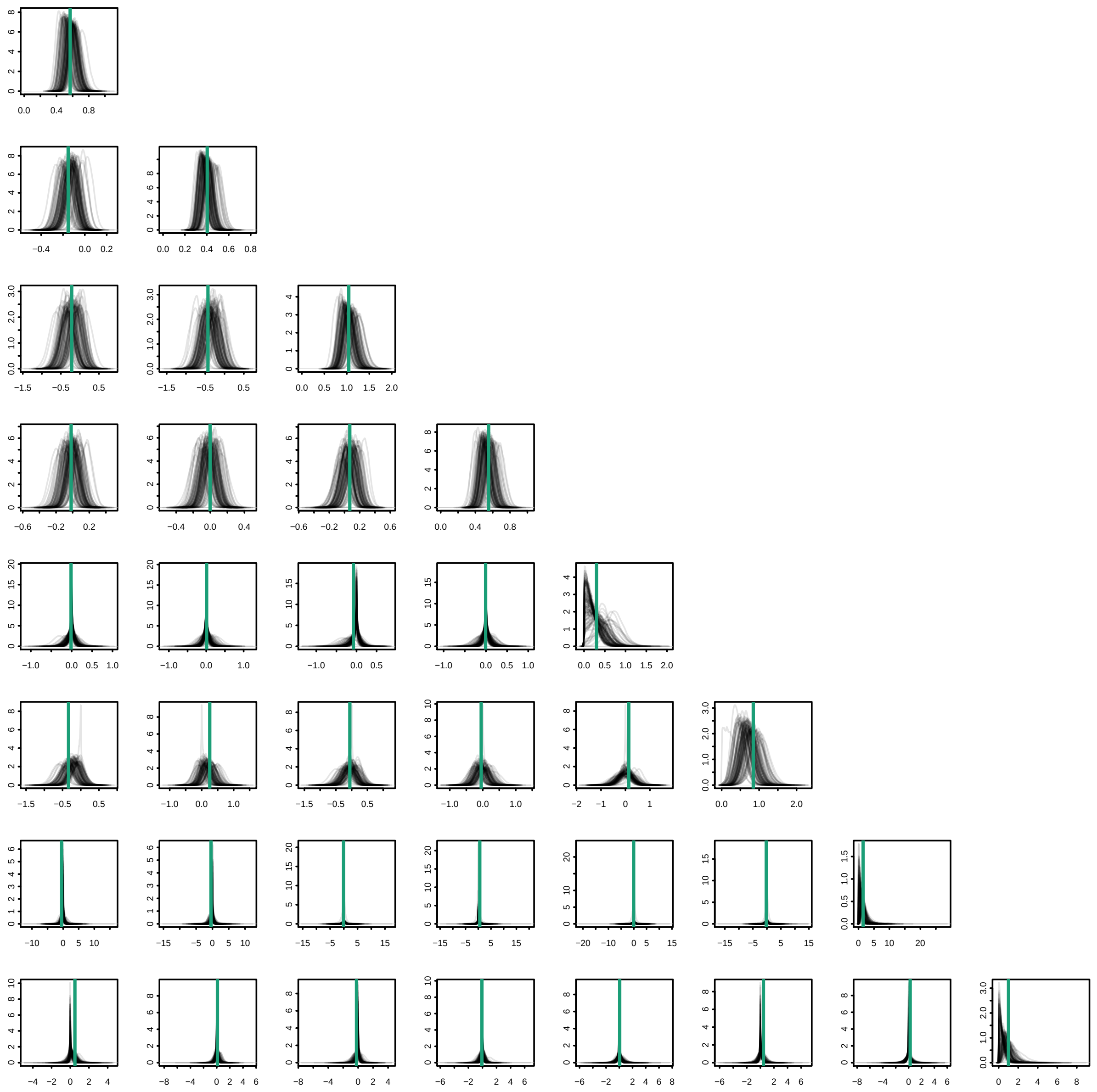

Figure S3: Posterior distributions (black lines) from 100 simulated data sets of estimated elements of the Cholesky factor of the error covariance matrix $\mathbf{\Sigma}$. Vertical green lines: true parameter values. Rows and columns are ilr components. Note that these coordinates have no simple interpretation in terms of the 9 taxa.

The long-tailed distributions and poorer performance of credible intervals for parameters associated with components 7 and 8 is probably a consequence of the number of observations for the taxa whose distributions they reflect. Component 7 is proportional to the log of the ratio of *Aurelia aurita* to the geometric mean of all taxa other than *Aurelia aurita* and colonial ascidians, and component 8 to the log of the ratio of colonial ascidians to all other taxa. Since *Aurelia aurita* and colonial ascidians were the least abundant taxa, with non-zero point counts in only 7 and 15 out of 125 stills respectively, it is not surprising that estimation of parameters describing their distributions is more difficult than for other taxa.

S5 Comparison with linear and cubic depth effects

We compared the performance of the model with quadratic depth effect described in the main text against that of models with linear and cubic depth effects. We fitted the linear and cubic models exactly as described for the quadratic model in the main text, with extra coefficient vector $\boldsymbol{\beta}_3$ describing the cubed depth effect in the cubic model, and with higher-order coefficient vectors set to $\mathbf{0}$ where necessary (e.g. in the quadratic model, $\boldsymbol{\beta}_3 = \mathbf{0}$).

We evaluated the ability of each model to predict new stills, rather than new points from existing stills. Since each still has its own intercept, it corresponds to a cluster in the language of hierarchical models. We therefore used a leave-one-out cross-validation procedure (e.g. Garthwaite et al., 2002, section 9.4) in which each still was omitted in turn, with loss function the Bayesian leave-one-cluster-out estimate of out-of-sample prediction error (expected log predictive density) $\mathrm{elpd}_{\mathrm{loco}}$:

$$\mathrm{elpd}_{\mathrm{loco}} = \sum_{i=1}^{m} \log f(\mathbf{y}_i | \mathbf{y}_{-i}),$$

where m is the number of stills and $f(\mathbf{y}_i | \mathbf{y}_{-i})$ is the posterior density of the ith still $\mathbf{y}_i$, given the data set $\mathbf{y}_{-i}$ in which the ith still is excluded (Vehtari et al., 2017). The required posterior

density is given by

$$f(\mathbf{y}_i|\mathbf{y}_{-i}) = \int f(\mathbf{y}_i|\boldsymbol{\theta})f(\boldsymbol{\theta}|\mathbf{y}_{-i})\,\mathrm{d}\boldsymbol{\theta}, \tag{S2}$$

where $\boldsymbol{\theta} = \{\boldsymbol{\beta}_0, \boldsymbol{\beta}_1, \boldsymbol{\beta}_2, \boldsymbol{\beta}_3, \boldsymbol{\Sigma}\}$ is set of regression coefficients and the covariance matrix $\boldsymbol{\Sigma}$, $f(\mathbf{y}_i|\boldsymbol{\theta})$ is the density of the ith still given parameters $\boldsymbol{\theta}$, and $f(\boldsymbol{\theta}|\mathbf{y}_{-i})$ is the posterior density of $\boldsymbol{\theta}$ given the dataset $\mathbf{y}_{-i}$. Because we want to predict a new still, we need to integrate over the distribution of still-specific intercepts ε_i. Thus

$$f(\mathbf{y}_i|\boldsymbol{\theta}) = \int f(\mathbf{y}_i|\boldsymbol{\beta}_0, \boldsymbol{\beta}_1, \boldsymbol{\beta}_2, \boldsymbol{\beta}_3, \varepsilon_i)f(\varepsilon_i|\boldsymbol{\theta})\,\mathrm{d}\varepsilon_i, \tag{S3}$$

where $\varepsilon_i \sim N(\mathbf{0}, \boldsymbol{\Sigma})$. Equation S3 is described as the marginal likelihood in Merkle et al. (2018), because it involves integrating out the still-specific parameters. Note that this is not the same as the full marginal likelihood that appears in the denominator of a posterior density.

We estimated the integral in Equation S3 using a classical Monte Carlo integration method (Robert and Casella, 2004, p.83), with 1000 draws from $N(\mathbf{0}, \boldsymbol{\Sigma})$ for each Stan iteration. We experimented with the quadrature method described in Merkle et al. (2018), but found that in 8 dimensions this required too many quadrature points to be feasible. We estimated the integral in Equation S2 using the mean from the values of $\boldsymbol{\theta}$ over 1000 Stan iterations for each data set $\mathbf{y}_{-i}$. We experimented with the Pareto-smoothed importance sampling method described in Vehtari et al. (2017), which often allows the expected log predictive density to be computed from a single fitted model, without separate fitting of the model to each leave-one-cluster-out data set. However, diagnostics suggested that the Pareto smoothing was not performing well enough to be reliable.

We computed standard errors of differences in $\mathrm{elpd}_{\mathrm{loco}}$ among models using the `compare()` function in the R package `loo`. These standard errors are approximate (Vehtari et al., 2017), suggesting that a fairly conservative interpretation of differences among models is sensible.

Plots of the expected relative abundances from each model suggest that the linear model is much worse than the quadratic (Figure S4, green vs black lines), but that the quadratic model is about as good as the cubic model (Figure S4, orange vs black lines). Differences in $\text{elpd}_{\text{loco}}$ confirm this: −1004 with standard error 64 between quadratic and linear, −49 with standard error 17 between cubic and quadratic.

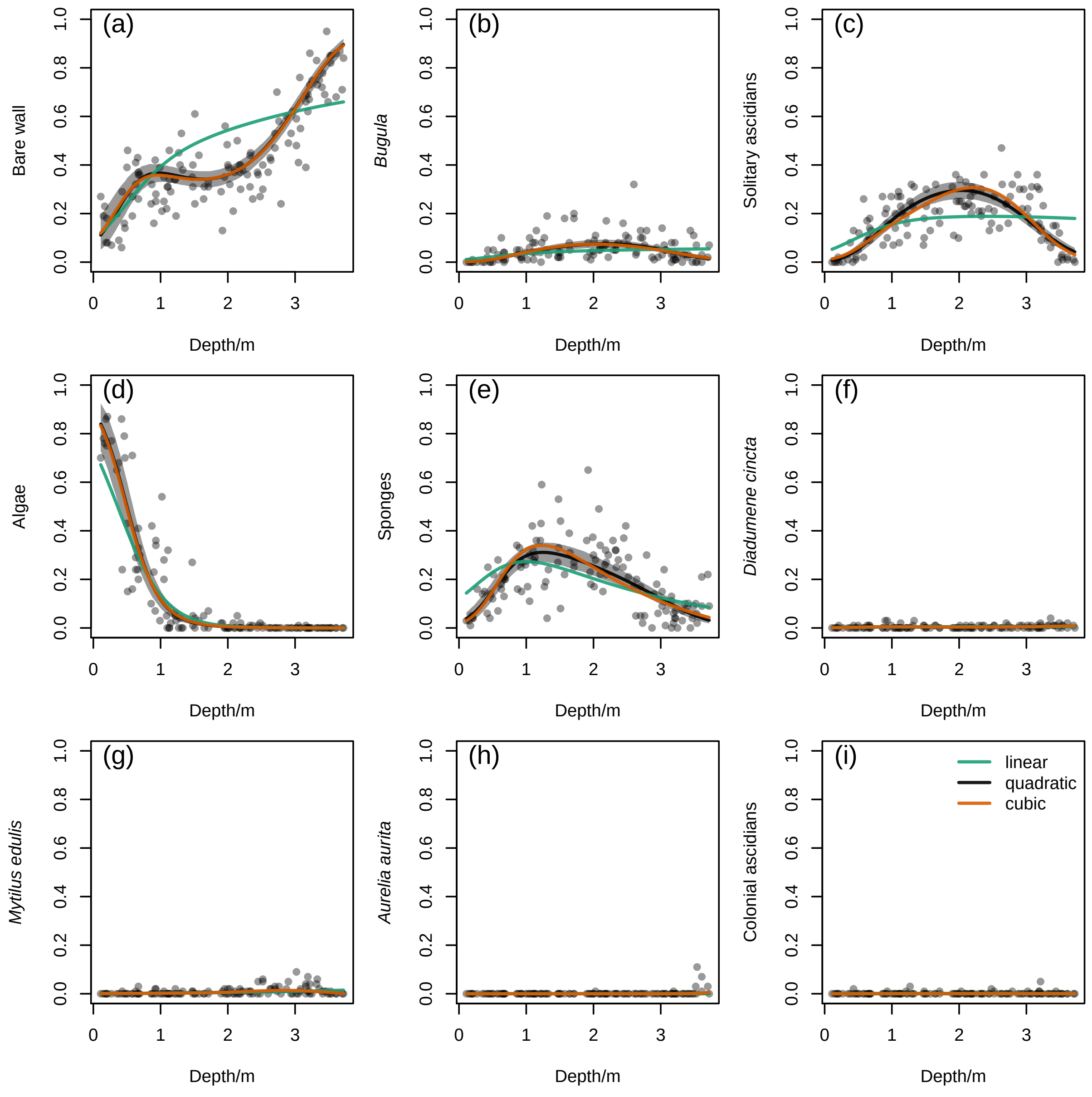

Figure S4: Estimated relationships between relative abundance and depth for bare wall and eight taxa, fitted using a quadratic model as described in the main text (black lines with grey 95% credible bands), a linear model (green lines) and a cubic model (orange lines). Circles are sample estimates of relative abundance from point counts.

S6 Construction of a meaningful ilr basis

Constructing a meaningful ilr basis is not too difficult for a fairly small number of taxa whose biology is well understood. In our case, the obvious choice for a first coordinate is to contrast bare wall with macroscopic organisms, since bare wall is likely to behave as a passive component in this system. A natural second coordinate is to contrast algae with animals. Algae are the only photosynthetic organisms in our study, and are thus expected to depend very strongly on light availability, which is likely to decrease rapidly with increasing depth. For the third coordinate, we contrasted predators (*Aurelia aurita* and *Diadumene cincta*) with filter-feeding animals. The filter-feeders are likely to rely heavily on phytoplankton, whose abundance is expected to decrease with depth. In contrast, the predators use tentacles to catch relatively large and highly motile zooplankton. For the fourth coordinate, we contrasted the two predators. For the fifth coordinate, we contrasted *Mytilus edulis*, which is the only mollusc and has a very high filtration rate, with the other filter feeders. For the sixth coordinate, we contrasted sponges with bryozoans and ascidians, and for the seventh coordinate, we contrasted bryozoans with ascidians. These two coordinates were chosen taxonomically, because taxonomic differences are likely to be associated with differences in responses to depth. Finally, we chose the eighth coordinate as the contrast between solitary and colonial ascidians, which have very different morphologies and are thus likely to respond differently to depth.

For each coordinate, we code the taxa on one side of the contrast as $+1$, those on the other side as -1, and those not involved in the contrast as 0. Rescaling the positive and negative elements to give a vector with zero sum, and then rescaling the entire vector to unit length, gives one column of the required basis matrix (Table S5). The columns are orthogonal by construction, because each contrast after the first is among taxa on only one side of the preceding contrasts.

Table S5: Biologically meaningful ilr basis, constructed as described in Section S6. Rows are taxa, and columns are basis vectors.

	1	2	3	4	5	6	7	8
Bare wall	$\frac{2\sqrt{2}}{3}$	0	0	0	0	0	0	0
Bugula	$-\frac{1}{3\sqrt{8}}$	$-\frac{1}{2\sqrt{14}}$	$-\frac{2}{\sqrt{70}}$	0	$-\frac{1}{2\sqrt{5}}$	$-\frac{\sqrt{3}}{6}$	$\frac{\sqrt{2}}{\sqrt{3}}$	0
Solitary ascidians	$-\frac{1}{3\sqrt{8}}$	$-\frac{1}{2\sqrt{14}}$	$-\frac{2}{\sqrt{70}}$	0	$-\frac{1}{2\sqrt{5}}$	$-\frac{\sqrt{3}}{6}$	$-\frac{1}{\sqrt{6}}$	$\frac{1}{\sqrt{2}}$
Algae	$-\frac{1}{3\sqrt{8}}$	$\frac{\sqrt{7}}{2\sqrt{2}}$	0	0	0	0	0	0
Sponges	$-\frac{1}{3\sqrt{8}}$	$-\frac{1}{2\sqrt{14}}$	$-\frac{2}{\sqrt{70}}$	0	$-\frac{1}{2\sqrt{5}}$	$\frac{\sqrt{3}}{2}$	0	0
Diadumene cincta	$-\frac{1}{3\sqrt{8}}$	$-\frac{1}{2\sqrt{14}}$	$\frac{\sqrt{5}}{\sqrt{14}}$	$-\frac{1}{\sqrt{2}}$	0	0	0	0
Mytilus edulis	$-\frac{1}{3\sqrt{8}}$	$-\frac{1}{2\sqrt{14}}$	$-\frac{2}{\sqrt{70}}$	0	$\frac{2}{\sqrt{5}}$	0	0	0
Aurelia aurita	$-\frac{1}{3\sqrt{8}}$	$-\frac{1}{2\sqrt{14}}$	$\frac{\sqrt{5}}{\sqrt{14}}$	$\frac{1}{\sqrt{2}}$	0	0	0	0
Colonial ascidians	$-\frac{1}{3\sqrt{8}}$	$-\frac{1}{2\sqrt{14}}$	$-\frac{2}{\sqrt{70}}$	0	$-\frac{1}{2\sqrt{5}}$	$-\frac{\sqrt{3}}{6}$	$-\frac{1}{\sqrt{6}}$	$-\frac{1}{\sqrt{2}}$

S7 Comparison with other approaches

We compared the results from our approach with those obtained by fitting overdispersed Poisson regressions with depth and depth squared as explanatory variables, using the `HMSC` package (Ovaskainen et al., 2017). We used the overdispersed Poisson response distribution, the default priors from the `HMSC` package, and a burnin period of 1000 iterations followed by 10000 sampling iterations, thinned to every 10th iteration. We then used the `predict()` method from `HMSC` to generate predicted counts, and transformed these into compositions using the `acomp()` method from the `compositions` package.

We also explored the use of multivariate linear models fitted to ilr-transformed count data with pseudocounts, with depth and depth squared as explanatory variables. Pseudocounts are necessary because the ilr transformation cannot be applied to data containing zero counts. We tested three different pseudocount values (Martín-Fernandez et al., 2011, Table 4.2): 1 (Laplace), 1/2 (Jeffreys) and 1/9 (Perks, with 9 components). These are well-known and widely-

used approaches, although other methods specifically designed for compositional data may be superior (Martín-Fernandez et al., 2011).

We also fitted a multivariate regression model by penalized likelihood, using the R package `glmnet` (Friedman et al., 2010). We chose the value of the regularization parameter λ by cross-validation, and used the `type.multinomial = "grouped"` option in order to ensure that all elements of a coefficient vector were either included or excluded together.

All approaches had similar behaviour for taxa with high relative abundance (Figure S5a-e). For taxa with low relative abundance (Figure S5f-i), predicted values from Laplace (orange lines) and Jeffreys (purple lines) pseudocounts and `HMSC` (cyan lines) were usually above the top of the 95% credible bands from our approach. Perks pseudocounts (pink lines) were generally closer to our approach. We would expect Laplace pseudocounts to result in overestimates of relative abundance for rare taxa. Although Laplace pseudocounts can be justified as a Bayesian estimator with equal prior probability for each taxon, such an approach gives a lot of weight to unseen taxa, and will become more problematic when the number of taxa is large (Manning and Schütze, 1999, p. 202). For these data, smaller pseudocounts appear better. The naive overdispersed Poisson regression approach taken here does not preserve the multinomial sums, so transformation of predicted values into compositions was needed afterwards. It is possible to transform the likelihood for a multinomial model into a product of independent Poisson likelihoods, and thus to fit a multinomial regression model using Poisson regression (Baker, 1994). Details for some models with random effects are discussed in Lee et al. (2017). It is therefore possible that the performance of `HMSC` on these data could be improved by coding new explanatory variables to properly represent a multinomial model. Both `HMSC` and `glmnet` gave predictions that responded more strongly than our approach to a small number of higher observed counts in the deeper images for *Mytilus edulis* and *Aurelia aurita* (Figure S5g, h: cyan and green lines respectively).

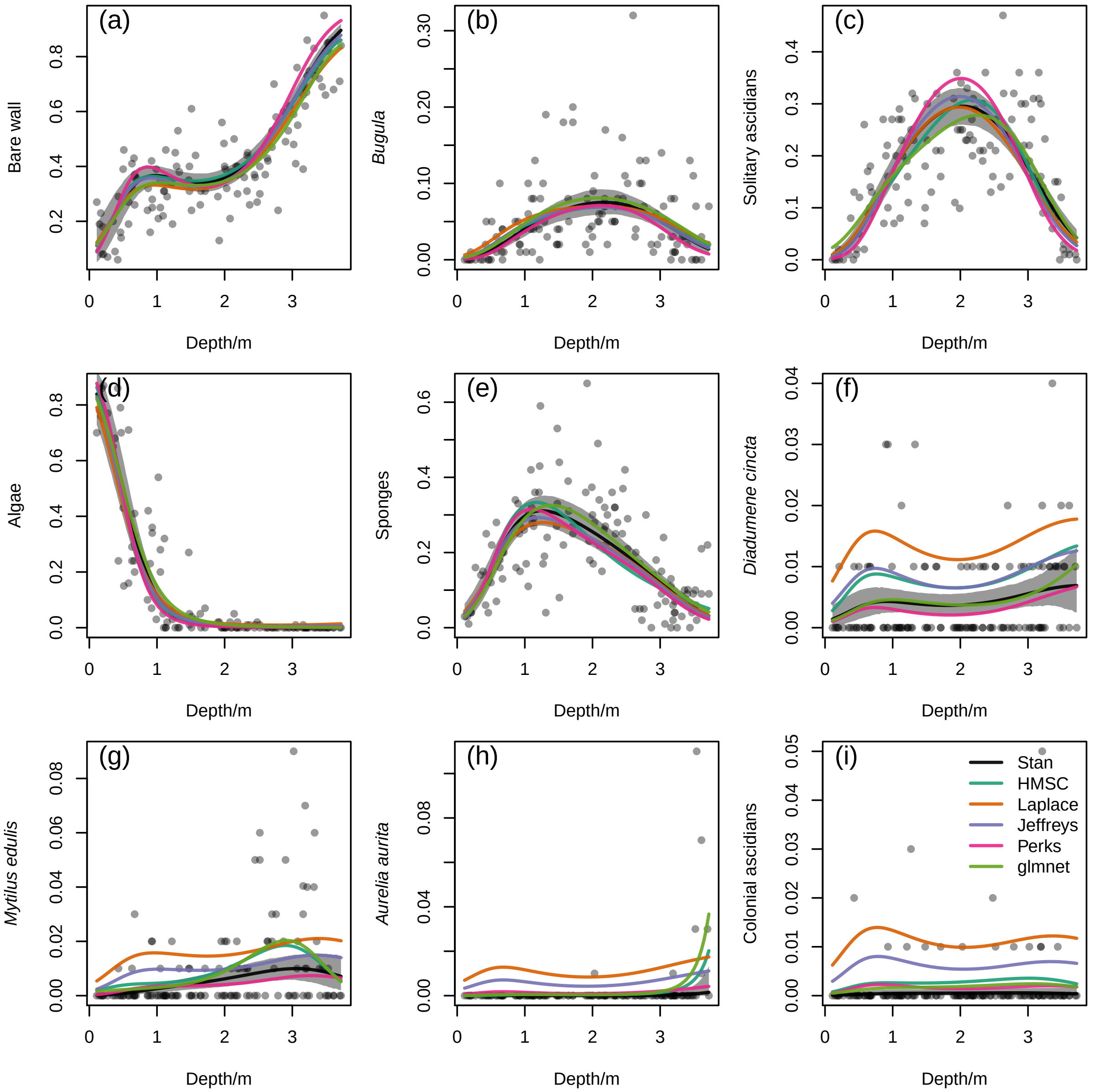

Figure S5: Estimated relationships between relative abundance and depth for bare wall and eight taxa, fitted using Stan as described in this paper (black lines with grey 95% credible bands), overdispersed Poisson regression in `HMSC` (Ovaskainen et al., 2017) transformed into compositions (cyan), multivariate linear models fitted to ilr-transformed count data with Laplace (orange), Jeffreys (purple) and Perks (pink) pseudocounts, and a multinomial regularized generalized linear model fitted using `glmnet` (green). Circles are sample estimates of relative abundance from point counts. Note that y-axis scales differ between panels in order to show detail for taxa with low relative abundances.

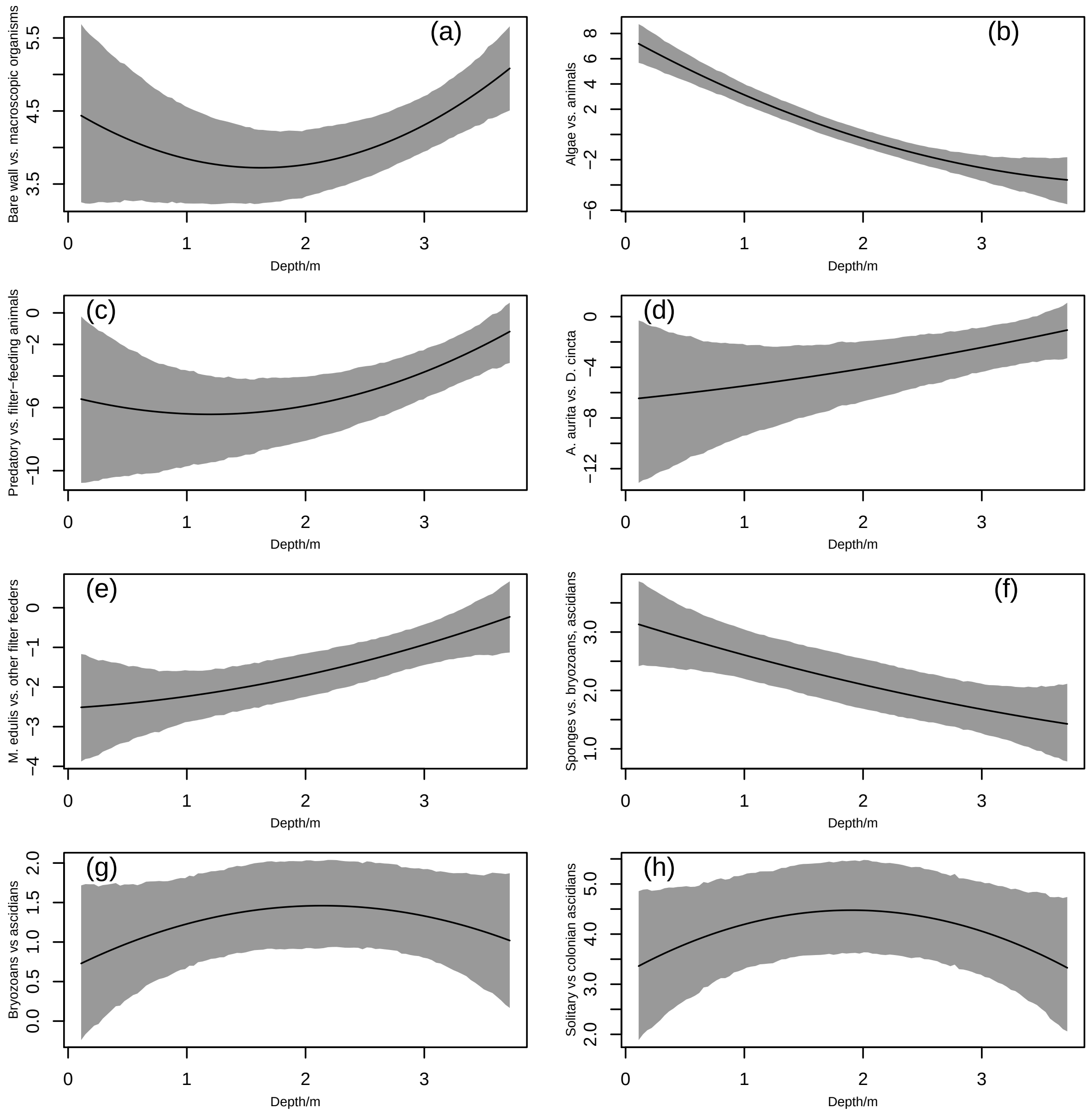

Figure S6: Estimated relationships between relative abundances in ilr coordinates (with the basis in Table S5) and depth. Grey bands are 95% HPD credible bands, and black lines are posterior means. Note the difference in y-axis scales among panels.

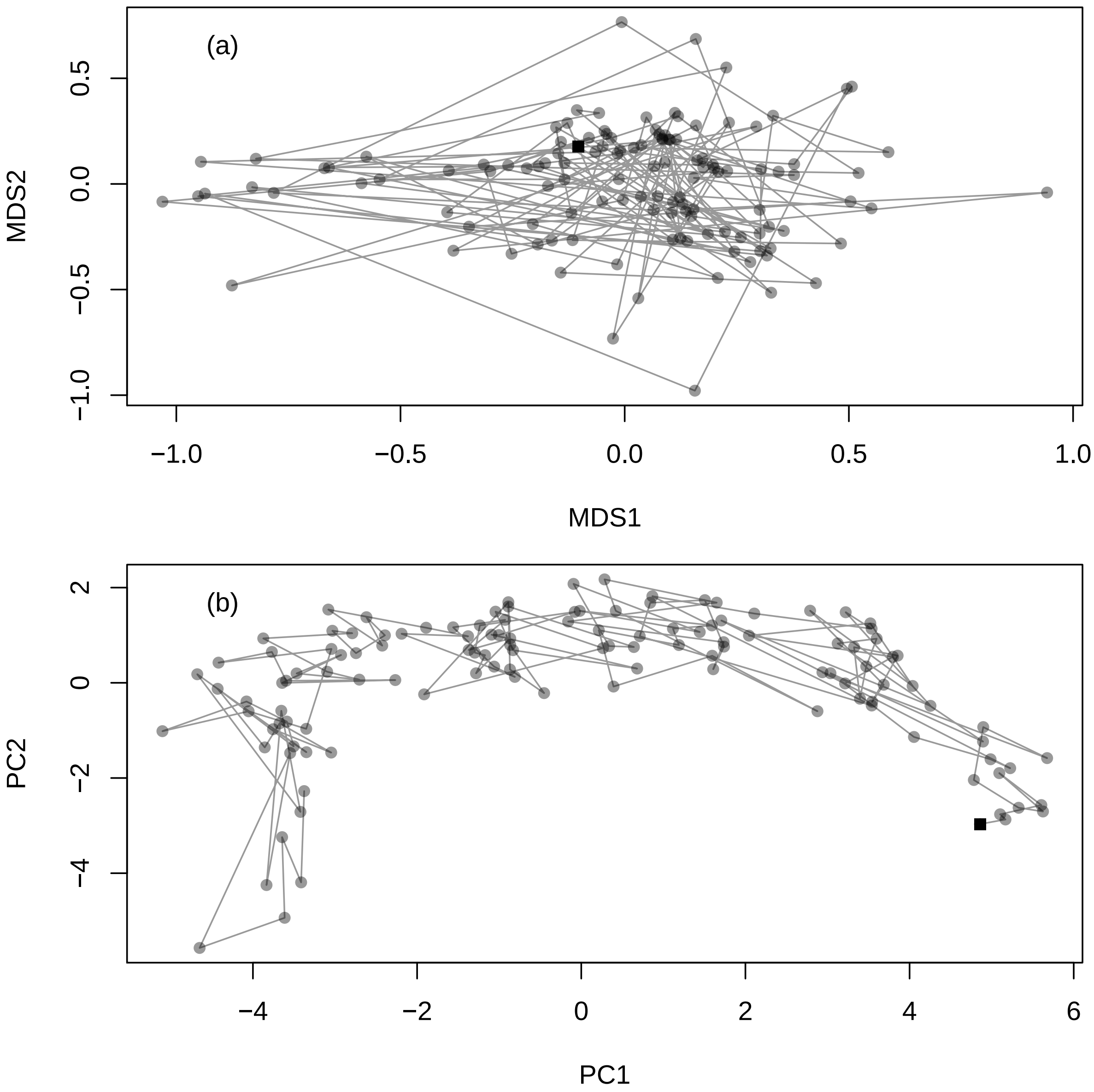

Figure S7: (a) Non-metric multidimensional scaling on raw counts (top), using `metaMDS()` with default options (square root transformation, Wisconsin double standardization, Bray-Curtis dissimilarity) in R package `vegan` (stress 0.19). (b) First two principal components of still-specific posterior mean predictions in ilr coordinates (cumulative 90% of variance explained). Lines connect stills in depth order, and the black square indicates the shallowest depth.

S8 Rate of change measured by Bray-Curtis dissimilarity

In order to show how the compositional data analysis approach changes the interpretation of environmental gradients for the marine community data, compared to the standard approach, we plotted Bray-Curtis dissimilarities between adjacent predicted relative abundances on a grid of 100 equally-spaced values of depth (Figure S8). These plots give rough estimates of the rate of change in relative abundance, as measured by Bray-Curtis dissimilarity. Under this approach, there appeared to be local maxima in the rate of change with respect to depth at approximately 0.5 m and at 3 m, separated by a local minimum and with lower rates of change shallower than 0.5 m and deeper than 3 m. The pattern is very different from that obtained using the compositional approach (Figure 5 in the main text), where there was a single local minimum in rate of change at approximately 2 m, and no local maxima.

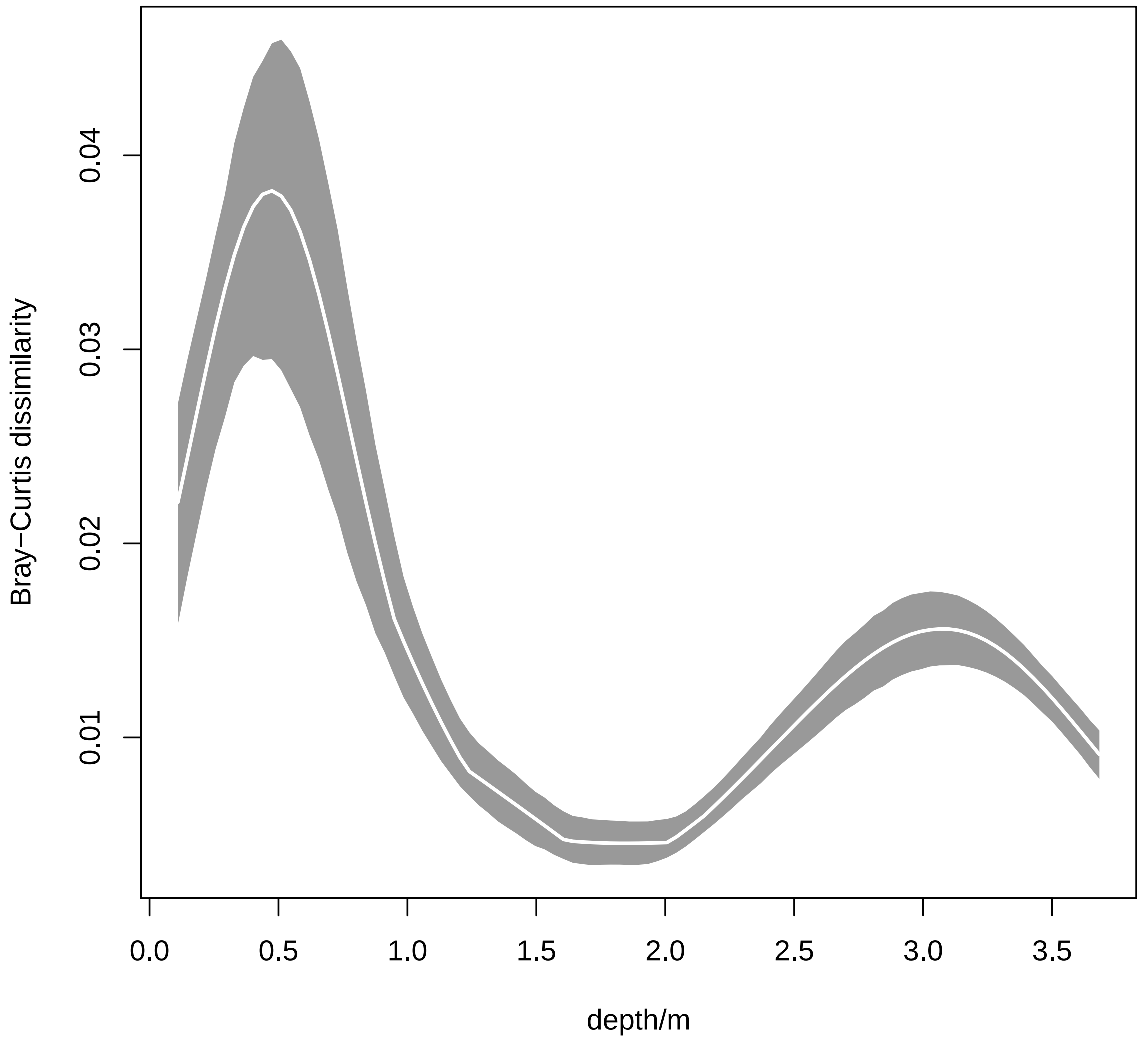

Figure S8: Bray-Curtis dissimilarity between adjacent predicted relative abundances on a grid of 100 equally-spaced values of depth (m). White line: posterior mean. Grey band: 95% HPD credible bands.

S9 Application to mite data

We fitted essentially the same model as that used in the main text to the `mite` data set in the R package `vegan`, described in Borcard et al. (1992). The data consist of counts of 35 taxa of mites in 70 *Sphagnum* moss cores. There are two quantitative non-spatial explanatory variables, substrate density ($\mathrm{g\,l^{-1}}$) and substrate water content ($\mathrm{g\,l^{-1}}$). Additional categorical explanatory variables and spatial coordinates are also available, but for simplicity, we did not use these in the model. For the same reason, we included only linear effects of substrate density and water content. We fitted the model using the same approach as for the marine community data described in the main text (although for speed, we ran only 1000 warmup and 1000 sampling iterations of each chain). We conditioned on the observed total counts, in order to study patterns in relative abundance, and used the same multinomial observation model as for the marine community data.

We would expect this to be a challenging data set to model, because it is high-dimensional and contains many rare taxa. For serious applications, it might be appropriate to use a more constrained covariance structure, and to use a hierarchical model for the regression coefficients. Nevertheless, the fitted model appeared plausible. For those taxa that were not rare, relationships with substrate density generally appeared fairly weak (Figure S9), compared to those for water content (Figure S10). In these figures, predictions were made with the explanatory variable that is not on the x-axis set to its mean value. In order to give a better impression of the fit of the model to data, Figure S11 shows posterior mean predicted relative abundances against observed relative abundances, with the values of explanatory variables that were actually observed. For most taxa, the points fall close to the line with slope 1 and y-intercept 0, indicating that the posterior mean predictions are plausible. However, there are a few taxa with many very low observed relative abundances and a few higher values (e.g. MPRO, Ceratoz1, HRUF, PPEL,

Lepidzts, Eupelops, Miniglmn: Figure S11h, r, v, x, ab, ac, ad respectively) for which the higher values were generally underpredicted.

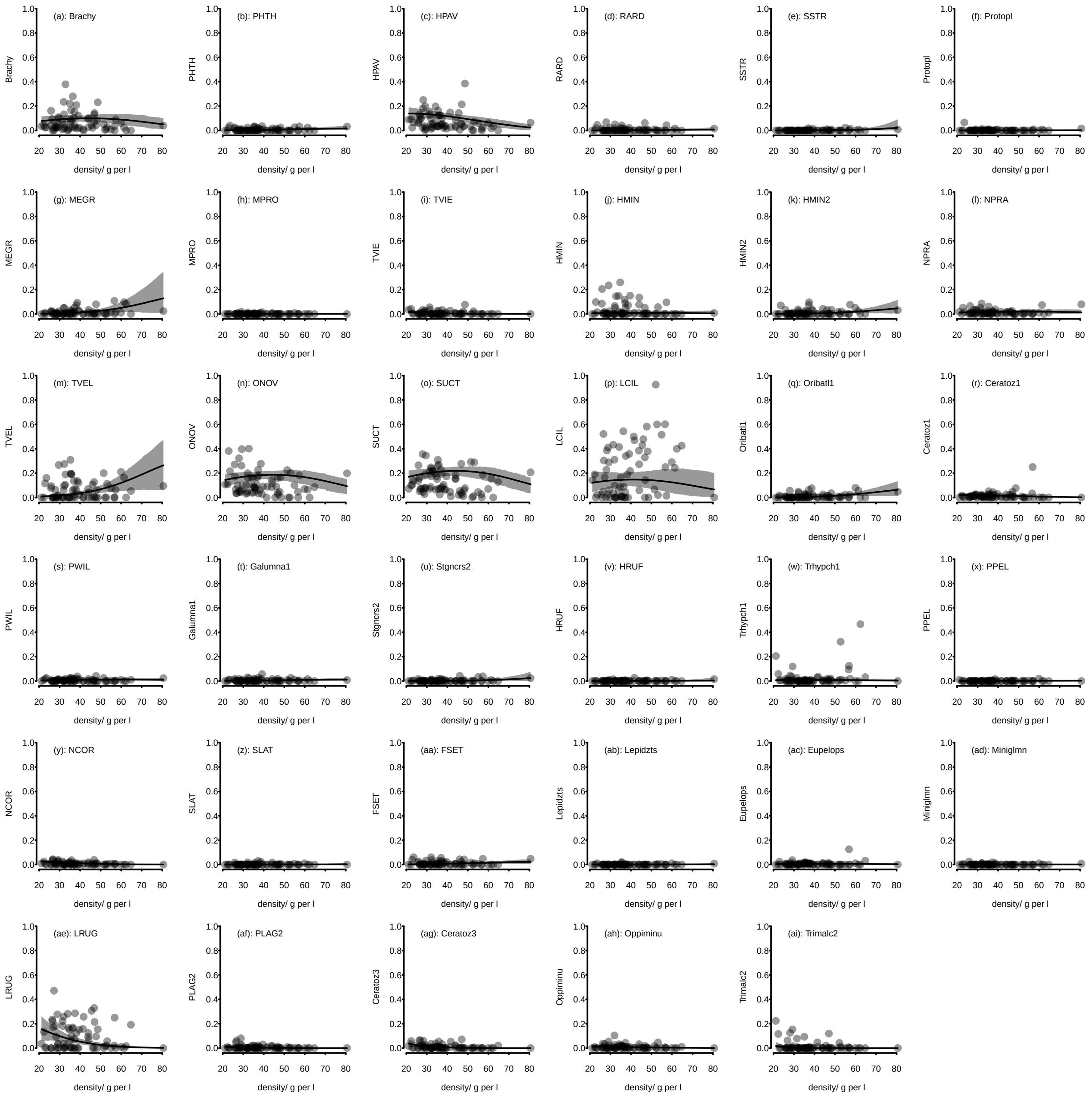

Figure S9: Relationships between relative abundance and substrate density ($\mathrm{g\,l^{-1}}$) for 35 mite taxa in 70 *Sphagnum* moss cores. Data from the `mite` data set in R package `vegan`, originally described in Borcard et al. (1992). Dots: observed relative abundances. Black lines: posterior mean predictions, with water content set to its mean value. Grey bands: 95% HPD credible band.

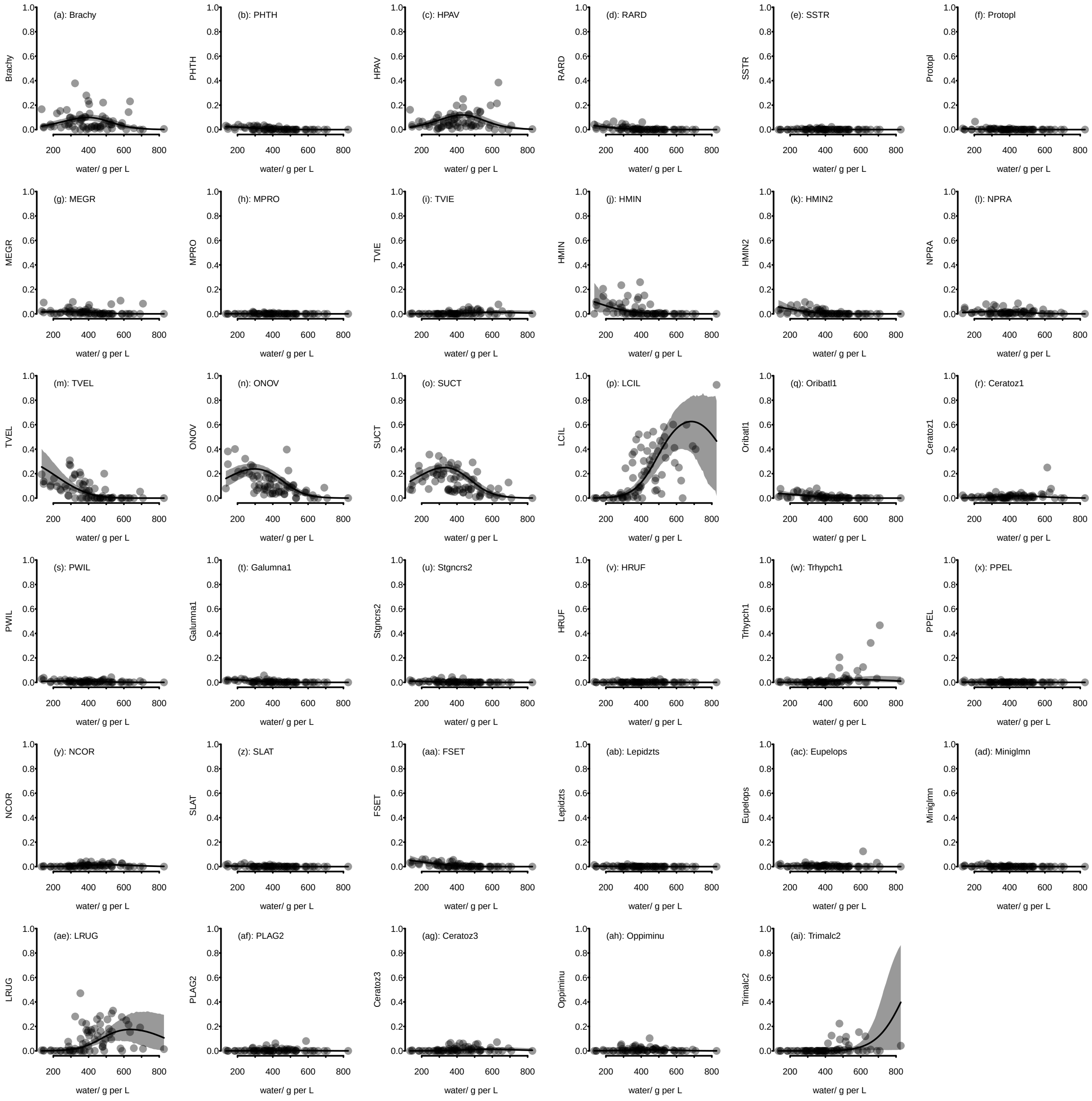

Figure S10: Relationships between relative abundance and substrate water content (g l^{-1}) for 35 mite taxa in 70 *Sphagnum* moss cores. Data from the `mite` data set in R package `vegan`, originally described in Borcard et al. (1992). Dots: observed relative abundances. Black lines: posterior mean predictions, with substrate density set to its mean value. Grey bands: 95% HPD credible band.

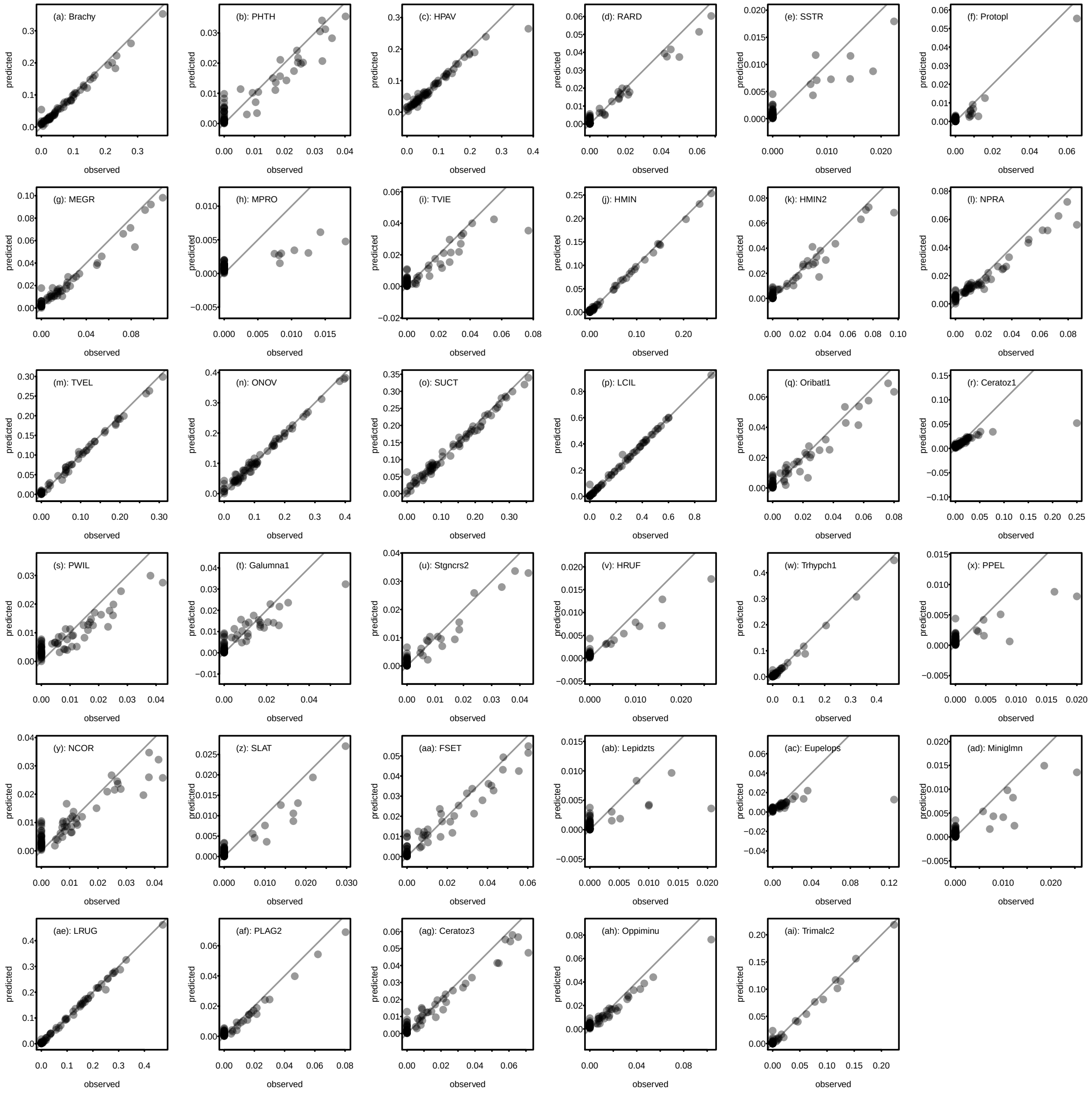

Figure S11: Posterior mean against observed relative abundances for 35 mite taxa in 70 *Sphagnum* moss cores. Data from the `mite` data set in R package `vegan`, originally described in Borcard et al. (1992). Each dot represents one core. Lines have slope 1, y-intercept 0.

As above (Section S8), we plotted Bray-Curtis dissimilarities between adjacent predicted relative abundances on a grid of 100 equally-spaced values of each of substrate water content (Figure S12a) and substrate density (Figure S12b). These plots give rough estimates of the rate of change in relative abundance, as measured by Bray-Curtis dissimilarity. Under a model with linear effects of each explanatory variable, the true rate of change of composition with respect to each explanatory variable, as measured using a perturbation-invariant distance, is a constant. However, the Bray-Curtis dissimilarity, which is not perturbation-invariant, changed in complicated ways with both water content (Figure S12a) and substrate density (Figure S12b). In particular, the apparent local maximum in rate of change at a water content of about $500\,\mathrm{g\,l^{-1}}$, and the apparent local minimum in rate of change at a substrate density of about $45\,\mathrm{g\,l^{-1}}$ are the kind of features that ecologists are likely to expend effort on explaining. Nevertheless, they are simply artefacts.

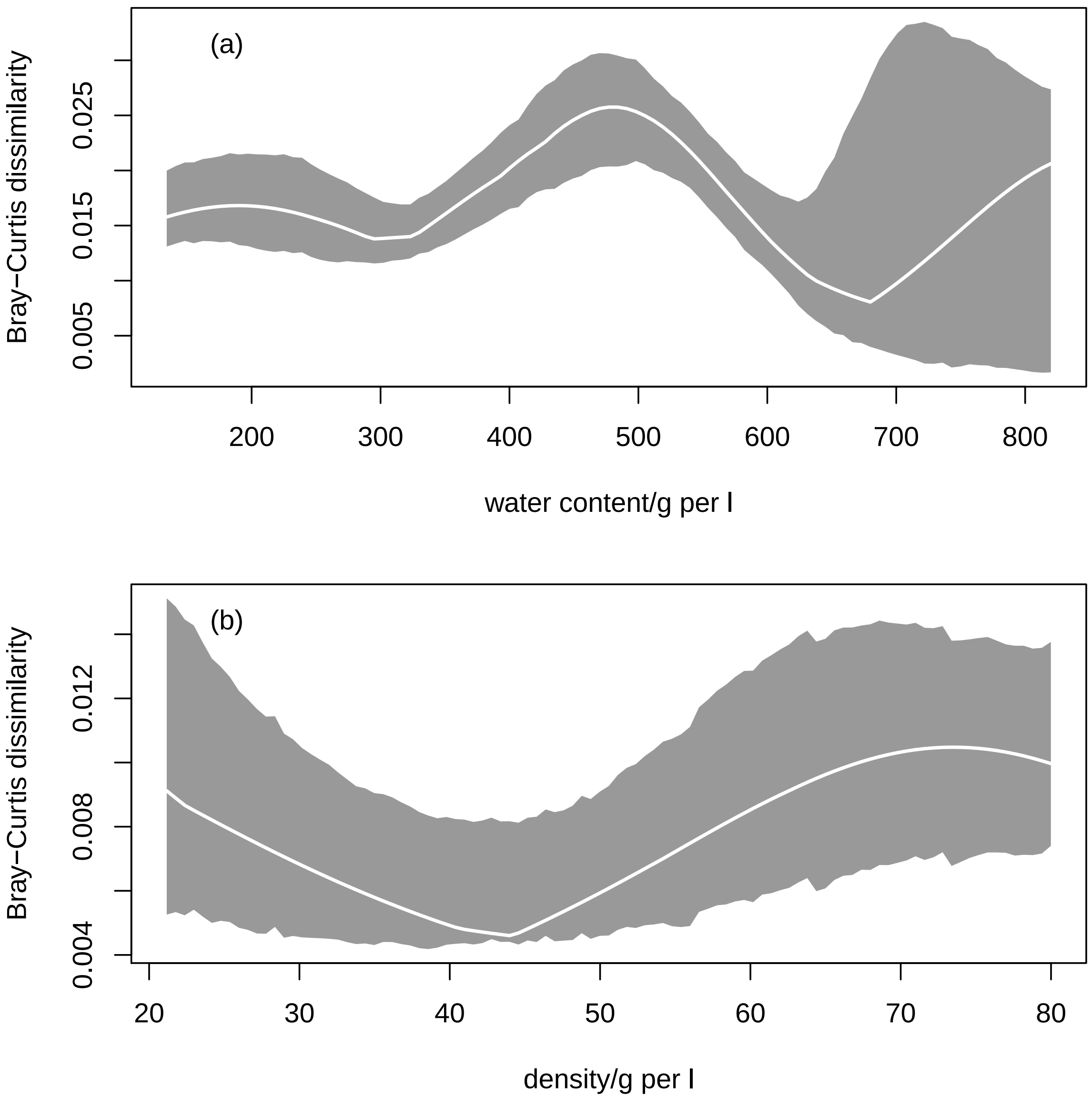

Figure S12: Bray-Curtis dissimilarities between adjacent predicted relative abundances on a grid of 100 equally-spaced values of (a) substrate water content ($g\,l^{-1}$) and (b) substrate density ($g\,l^{-1}$). In each case, the explanatory variable not on the x-axis was set to its mean value. White lines: posterior means. Grey envelopes: 95% HPD credible bands.

References

Aitchison, J. (1986). *The statistical analysis of compositional data*. Chapman and Hall, London.

Aitchison, J. (1992). On criteria for measures of compositional difference. *Mathematical Geology*, 24:365–379.

Baker, S. G. (1994). The multinomial-Poisson transformation. *The Statistician*, 43:495–504.

Billheimer, D., Guttorp, P., and Fagan, W. F. (2001). Statistical interpretation of species composition. *Journal of the American Statistical Association*, 96:1205–1214.

Borcard, D., Legendre, P., and Drapeau, P. (1992). Partialling out the spatial component of ecological variation. *Ecology*, 73:1045–1055.

Crowe, M. J. (1994). *A history of vector analysis: the evolution of the idea of a vectorial system*. Dover Publications, Inc., New York.

Dorier, J.-L. (1995). A general outline of the genesis of vector space theory. *Historia Mathematica*, 22:227–261.

Fraleigh, J. B. and Beauregard, R. A. (1995). *Linear algebra*. Addison-Wesley, Reading, Massachusetts, third edition.

Friedman, J., Hastie, T., and Tibshirani, R. (2010). Regularization paths for generalized linear models via coordinate descent. *Journal of Statistical Software*, 33:1–22.

Garthwaite, P. H., Jolliffe, I. T., and Jones, B. (2002). *Statistical Inference*. Oxford University Press, Oxford, second edition.

Hoffman, M. D. and Gelman, A. (2014). The No-U-Turn Sampler: Adaptively setting path lengths in Hamiltonian Monte Carlo. *Journal of Machine Learning Research*, 15:1351–1381.

Kennedy, Jr., W. J. and Gentle, J. E. (1980). *Statistical computing*. Marcel Dekker, New York.

Lee, J. Y. L., Green, P. J., and Ryan, L. M. (2017). On the “Poisson trick” and its extensions for fitting multinomial regression models. *unpublished*, arXiv:1707.08538v1.

Lewandowski, D., Kurowicka, D., and Joe, H. (2009). Generating random correlation matrices based on vines and extended onion method. *Journal of Multivariate Analysis*, 100:1989–2001.

Manning, C. D. and Schütze, H. (1999). *Foundations of statistical natural language processing*. The MIT Press, Cambridge, Massachusetts.

Martín-Fernandez, J. A., Palarea-Albaladejo, J., and Olea, R. A. (2011). Dealing with zeros. In Pawlowsky-Glahn, V. and Buccianti, A., editors, *Compositional data analysis: theory and applications*, pages 43–58. John Wiley & Sons, Ltd, Chichester.

Merkle, E. C., Furr, D., and Rabe-Hesketh, S. (2018). Bayesian model assessment: use of conditional vs marginal likelihoods. *unpublished*, arXiv:1802.04452v1.

Oksanen, J., Blanchet, F. G., Friendly, M., Kindt, R., Legendre, P., McGlinn, D., Minchin, P. R., O’Hara, R. B., Simpson, G. L., Solymos, P., Stevens, M. H. H., Szoecs, E., and Wagner, H. (2017). *vegan: Community Ecology Package*. R package version 2.4-3.

OpenStreetMap contributors (2018). Planet dump retrieved from https://planet.osm.org . `https://www.openstreetmap.org`. Accessed 15 July 2018.

Ovaskainen, O., Tikhonov, G., Norberg, A., Blanchet, F. G., Duan, L., Dunson, D., Roslin, T., and Abrego, N. (2017). How to make more out of community data? a conceptual framework and its implementation as models and software. *Ecology Letters*, 20:561–576.

R Core Team (2017). R: A language and environment for statistical computing. `https://www.R-project.org/`.

Robert, C. and Casella, G. (2004). *Monte Carlo Statistical Methods*. Springer, New York, second edition.

Spencer, M. (2015). Size change, shape change, and the growth space of a community. *Journal of Theoretical Biology*, 369:23–41.

Stan Development Team (2017). *Stan Modeling Language Users Guide and Reference Manual, Version 2.16.0.*

Vehtari, A., Gelman, A., and Gabry, J. (2017). Practical Bayesian model evaluation using leave-one-out cross-validation and WAIC. *Statistics and Computing*, 27:1413–1432.
